# Epithelial-Predominant Synovial Sarcoma With a Deceptive Neuroendocrine Phenotype

**DOI:** 10.7759/cureus.82988

**Published:** 2025-04-25

**Authors:** Rayan Rammal, Raja Seethala, Vikram Gorantla, John Skaugen, Aatur D Singhi, Rana Naous

**Affiliations:** 1 Pathology, University of Pittsburgh Medical Center, Pittsburgh, USA; 2 Oncology, University of Pittsburgh Medical Center, Pittsburgh, USA

**Keywords:** biphasic, differentiation, epithelial-predominant, neuroendocrine, synovial sarcoma

## Abstract

Synovial sarcoma (SS) is a tumor of uncertain lineage with a characteristic mixture of spindled and epithelial components that is subtyped as biphasic when both spindle and epithelial components are present and as monophasic when composed solely of spindle cells. Epithelial-predominant SS is extremely rare, and neuroendocrine differentiation within this component would be exceptional. Prominent ossification is another uncommon feature in SS, thought to portend a better prognosis. The hallmark genomic alteration in SS is t(X;18;p11;q11), resulting in the formation of an oncogenic fusion gene, usually *SS18(SYT):: SSX1* or *SS18::SSX2* and rarely *SS18::SSX4. *We herein describe a case of an epithelial-predominant SS arising in the right inguinal soft tissue of a 62-year-old woman. The mass is discovered incidentally on imaging and is peripherally calcified and stable during the first four years of follow-up, but later doubles in size. Microscopic examination reveals an epithelial-predominant malignant neoplasm forming glands and solid sheets, embedded in a variably cellular but mostly fibrotic-looking stroma. Prominent ossification is seen peripherally. The epithelial component is high-grade and shows neuroendocrine differentiation, further confirmed by positivity for synaptophysin and INSM-1. *SS18::SSX4 *is detected by RNA sequencing. Break-apart fluorescence in situ hybridization (FISH) performed separately on the epithelial and stromal components confirms an SS18 rearrangement, with the epithelial component additionally showing amplification of the region corresponding to the 5' end of SS18*. *We speculate that the latter finding could have played a role in the neuroendocrine transformation and progression of this tumor. Herein, we present a rare case of epithelial-predominant SS with unusual neuroendocrine differentiation mimicking a carcinoma.

## Introduction

Synovial sarcoma (SS) is commonly described as a spindle cell sarcoma with a variable degree of epithelial differentiation. The majority of SSs arise in the deep soft tissue of the extremities, often juxta-articular, and usually present in young adults less than 50 years old. SS shows a specific chromosomal translocation, t(X;18;p11;q11), resulting in the formation of an oncogenic fusion gene, usually either SS18(SYT)::SSX1 or SS18::SSX2 and rarely SS18::SSX4 [1]. So far, there is no evidence of any correlation between the different fusion partners (SSX1, SSX2, and SSX4) and SS tumor morphology. SSs are classified as biphasic (spindle and epithelial components) or monophasic (most cases). When gland-forming, the epithelial cells are cuboidal or columnar, with ovoid vesicular nuclei and more abundant pale eosinophilic cytoplasm relative to the darker/blue spindle cells. Focally, the glandular component may predominate and even be confused with an adenocarcinoma. However, a scant spindle cell component is typically still present [1]. Poorly differentiated areas are much more common in the elderly [1,2] and usually portend a worse prognosis [3]. These areas can be comprised of small round hyperchromatic cells, mimicking a small round blue cell tumor, or show a fascicular spindle cell or epithelioid morphology [1]. SS with neuroendocrine differentiation is an exceedingly rare phenomenon with only one case of predominantly epithelial-type SS with “overwhelming” neuroendocrine differentiation, in a young patient, reported recently [4]. We herein report a case of epithelial-predominant SS with neuroendocrine differentiation in a 62-year-old patient mimicking a metastatic carcinoma. The case we present not only is unusual in its morphology but also shows unique molecular findings, including the presence of a hitherto undescribed amplification of the 5’ end of the SS18 gene.

## Case presentation

Clinical presentation

**Figure 1:**
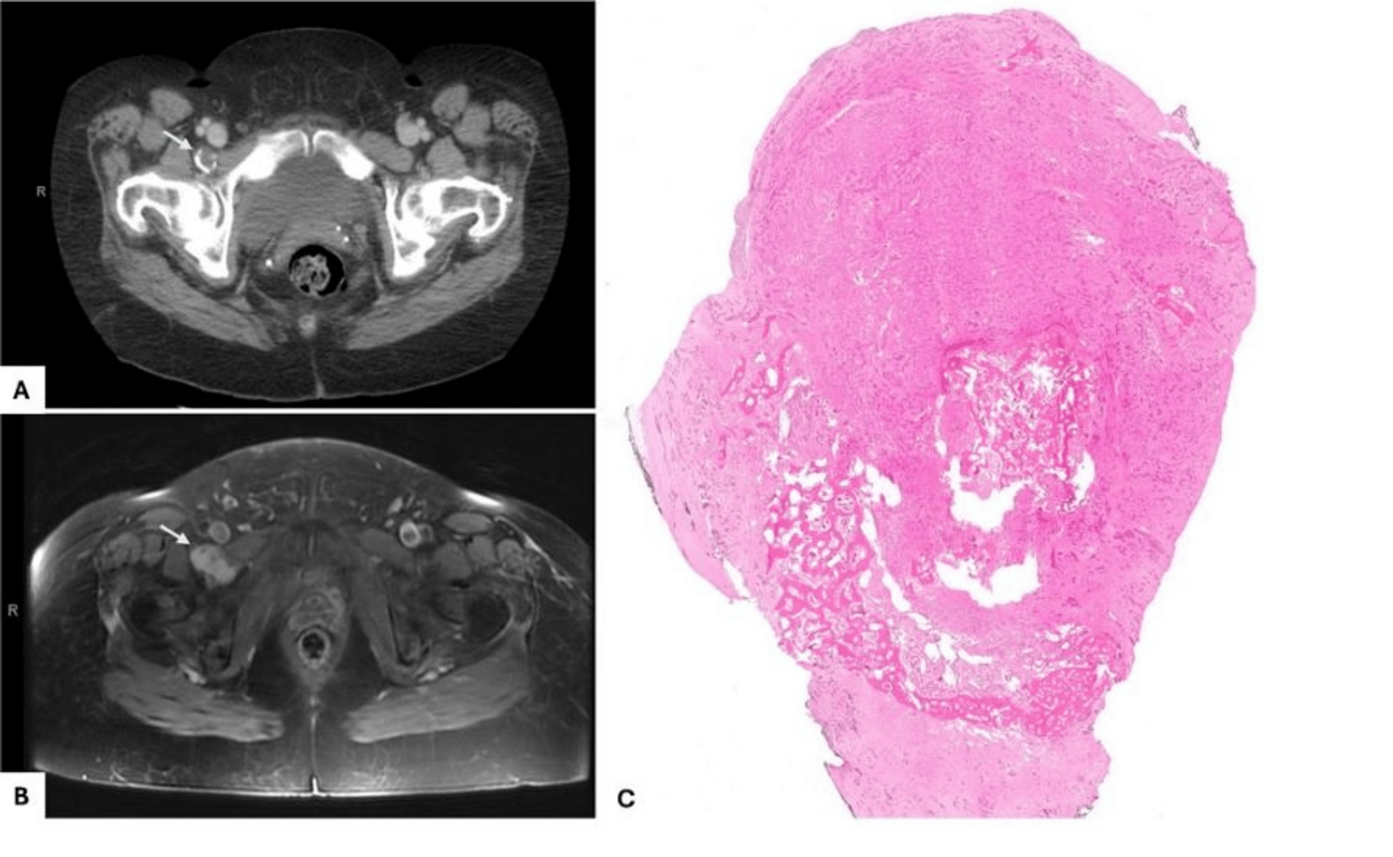
Imaging findings and scanning magnification microscopic examination (A) CT scan of the abdomen and pelvis with contrast showing a well-demarcated peripherally calcified lesion (arrow), described as a “lymph node,” anterior to the right hip joint and posterior to the right external iliac vein, measuring 1.8 x 1.5 cm. (B) MRI scan six years later demonstrates a macrolobulated and smoothly marginated mass, with faint enhancement, measuring 4.0 x 3.7 x 2.0 cm, dorsal to the right common iliac artery and vein (arrow). (C) On low-power microscopic examination, the mass is fairly circumscribed and shows prominent ossification, accentuated peripherally (0.5x, hematoxylin and eosin stain).

A 62-year-old female patient who initially presented for right groin pain after a motor vehicle accident was incidentally found to have a calcified “lymph node” anterior to the right hip joint and posterior to the external iliac vein, measuring 1.8 x 1.5 cm, on a CT scan (Figure 1A).

**Figure 2:**
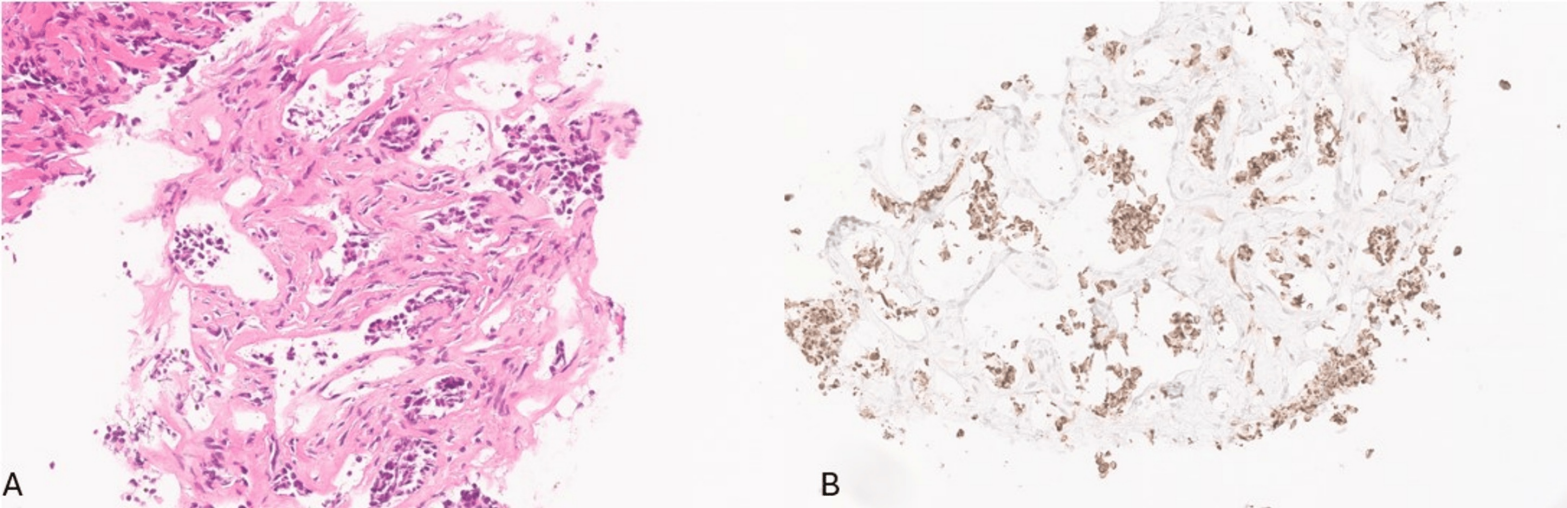
Original ultrasound-guided fine-needle aspiration (FNA) biopsy findings (A) FNA biopsy demonstrating epithelial cells forming glands or in loose clusters and set in a fibrotic mildly cellular stroma (200x, hematoxylin and eosin (H&E)). (B) Cytokeratin AE1/AE3 immunostain highlighting the epithelial cells.

The mass was initially stable for four years after the initial detection; however, it started doubling in size, and within the last two years, it measured 4.3 x 3.7 x 2.0 cm as per MRI (Figure 1B). Ultrasound-guided fine-needle aspiration (FNA) biopsy (FNAB) was performed and was reported as metastatic carcinoma (Figure 2A), with positivity for CKAE1/3 (Figure 2B). CK7, GATA-3, and ER were also reported to be positive; however, slides were not available for review.

A mammogram (performed at an outside institution, images not available) was negative. A PET scan (performed at an outside institution, images not available) showed no additional fludeoxyglucose (FDG)-avid disease regionally or distantly, aside from the mass in question. Given the lack of any obvious source of malignancy by imaging, the patient underwent excision of her right inguinal soft tissue mass for diagnostic and therapeutic purposes.

Macroscopic and microscopic examination

**Figure 3:**
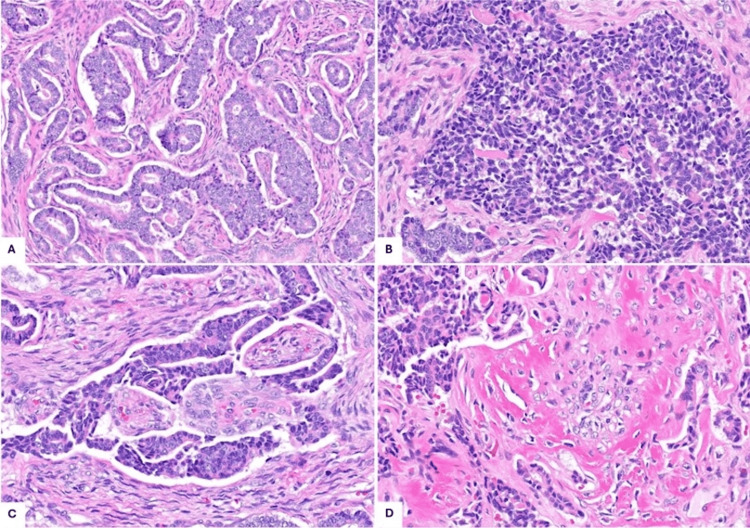
Histologic appearance (A) Malignant glands surrounded by a predominantly fibrotic-appearing stroma (100x, hematoxylin and eosin (H&E)). (B) Epithelial cells growing in solid sheets with a “salt and pepper” chromatin pattern (200x, H&E). (C) The stromal cells are more plump focally and arranged in vague whorls (200x, H&E). (D) Bone formation/osteoid deposition is noted in the tumor (200x, H&E).

The surgically excised tumor was received in two partly calcified lobulated fragments (2.7 x 2.2 x 1.3 cm and 4.7 x 4.7 x 2.4 cm) with glistening, tan-yellow, tan-white, and focally hemorrhagic cut surfaces. Ossification was peripherally accentuated (Figure 1C). Higher magnification revealed a malignant proliferation with gland/tubule formation set in a variably cellular, predominantly fibrotic-looking stroma (Figure 3A).

The epithelial cells had a small amount of pale eosinophilic cytoplasm and rather fine chromatin with pinpoint nucleoli. Areas with solid sheets, in which the nuclei showed a more prominent neuroendocrine or “salt and pepper” appearance (Figure 3B), were also seen. The mitotic rate was up to 10 mitoses per 10 high-power fields (HPFs), and foci of necrosis were present. The stroma was mostly densely collagenous with bland, wavy, thin, spindled nuclei. Focally, the stroma revealed more plump spindle cells with vague whorling (Figure 3C). Calcification as well as extensive ossification/bone formation was noted within the tumor (Figure 3D). There was no lymphoid tissue or lymph node architecture identified. The tumor extended to the resection margin, and vascular invasion was noted.

Ancillary studies and immunohistochemical stains

A panel of immunohistochemical stains was performed (see Table 1 for detailed results).

**Table 1:** Information on antibodies and histochemical stains employed in this study

Antibody	Source	Clone	Dilution	Result
EMA	Cell Marque	E29	Pre-dilute	Positive in the epithelial component
Cytokeratin 7	Ventana	SP52	Pre-dilute	Positive in the epithelial component
GATA-3	Biocare Medical	L50-823	Pre-dilute	Positive, patchy weak
ER	Ventana	SP1	Pre-dilute	Positive, weak in scattered epithelial cells
PR	Ventana	16	Pre-dilute	Negative
TRPS-1	Bio SB	EP392	1:1,000	Positive in the stromal component
TTF-1	Ventana	8G7G3/1	Pre-dilute	Negative
PAX-8	Cell Marque	SP348	Pre-dilute	Negative
SALL-4	Ventana	6E3	Pre-dilute	Negative
Synaptophysin	Ventana	SP11	Pre-dilute	Positive in the epithelial component
INSM-1	Cell Marque	MRQ-70	Pre-dilute	Positive in the epithelial component
GATA-6	Cell Signaling	D61E4	1:200	Positive in both components, stronger in the epithelial component
NKX2.2	Abcam	EPR14638	1:3,000	Positive in the epithelial component
TLE-1	Cell Marque	1F5	Pre-dilute	Positive: diffuse in the epithelial component; focal in the spindle component
BCL-2	Leica	Bcl-2/100/D5	Pre-dilute	Positive in the epithelial component
CDX-2	Biogenex	CDX2-88	1:200	Negative
Chromogranin	Ventana	LK2H10	Pre-dilute	Negative
ERG	Ventana	EPR3864	Pre-dilute	Negative
WT-1	Ventana	6F-H2	Pre-dilute	Negative
Calretinin	Ventana	SP65	Pre-dilute	Negative
SOX-10	Biocare Medical	BC34	Pre-dilute	Negative
S100	Dako	-	Pre-dilute	Negative
CD10	Leica	56C6	Pre-dilute	Negative
b-Catenin	Dako	B-Catenin-1	1:250	Negative
NUT	Abcam	Polyclonal	1:100	Negative
AR	Dako	AR441	1:100	Negative
P53	Ventana	DO-7	Pre-dilute	Wild-type
PDX-1	Abcam	EPR22002	1:100	Negative
POU2F3	Cell Signaling	E5N2D	1:500	Negative
MASH-1	BD Pharmingen	24B72D11.1	1:50	Negative
GFAP	Ventana	EP672Y	Pre-dilute	Negative
NeuroD	Abcam	EPR17084	1:100	Negative
BRG-1	Abcam	EPNCIR111A	1:25	Retained
INI-1	BD Transduction Laboratories	25/BAF47	1:50	Retained
Ki-67	Dako	MIB-1	1:75	11.6% (by supervised image analysis)

**Figure 4:**
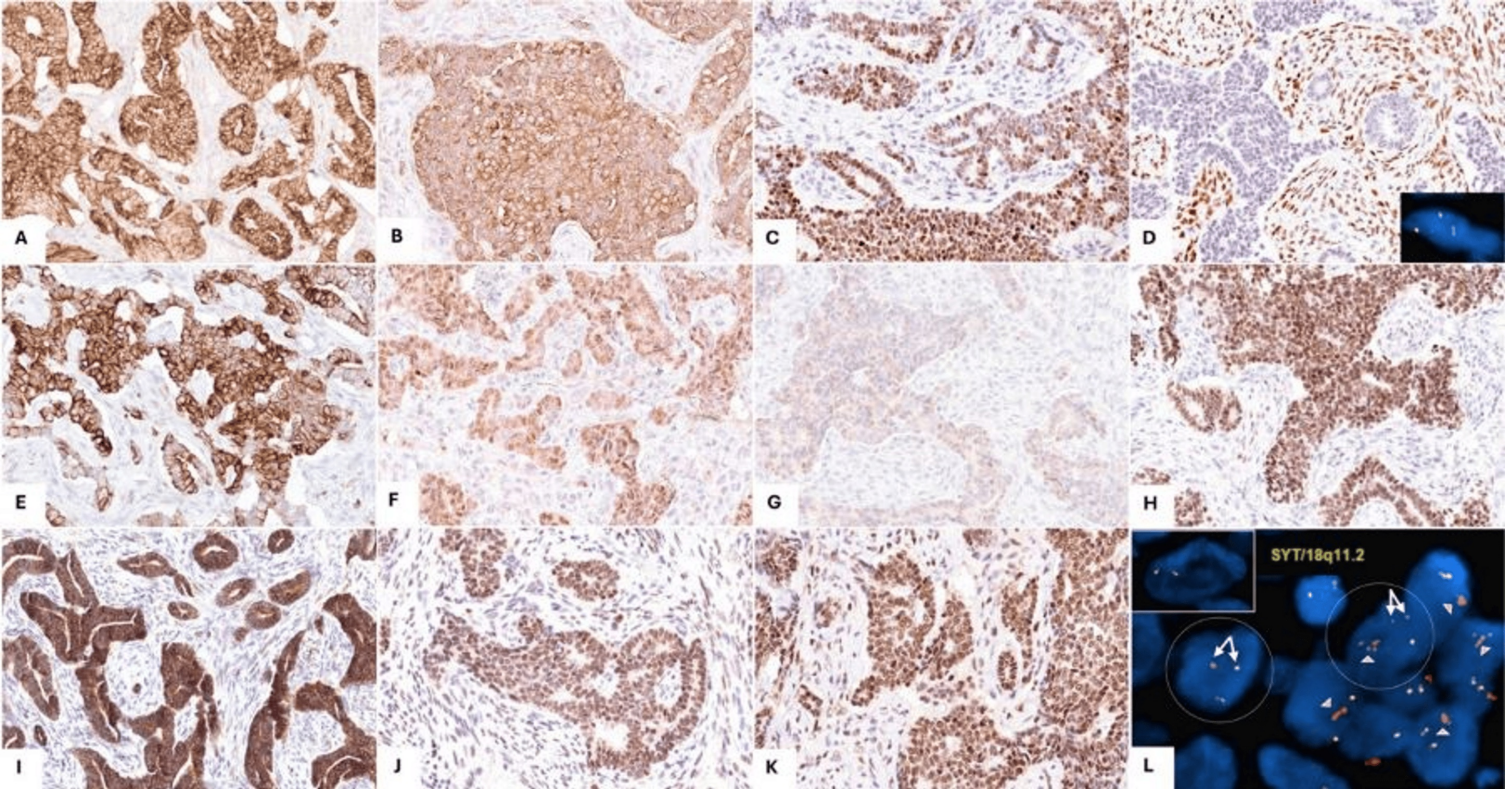
Ancillary studies The malignant epithelial cells are diffusely positive for (A) EMA, (B) synaptophysin, and (C) INSM-1. (D) TRPS-1 is positive in the stroma and negative in the epithelium; break-apart fluorescence in situ hybridization (FISH) is positive for SS18(SYT) rearrangement (inset). The epithelial component is also positive for (E) CK7, (F) GATA-3 (weak), (G) ER (rare weak positivity), and (H) NKX2.2 (diffuse strong). (I) Bcl-2, (J) GATA-6, and (K) TLE-1 are positive in both components but stronger and more diffuse in the epithelium compared to the stroma. (L) Break-apart FISH shows SS18(SYT) rearrangement (encircled translocated cells contain one separate green and one separate orange signal (arrows) in addition to one fused signal) as well as amplification of the orange signal (arrowheads), corresponding to the 5’ region of SS18. The inset (upper left corner) represents a control cell with two fused signals.

The epithelial component was diffusely positive for EMA (Figure 4A), synaptophysin (Figure 4B), and INSM-1 (Figure 4C). TRPS-1 was diffusely positive in the stroma but negative in the epithelium (Figure 4D). The epithelium was additionally positive for CK7 (Figure 4E), GATA-3 (Figure 4F), and ER (in scattered cells) (Figure 4G). NKX2.2 was diffusely and strongly positive in the epithelium and essentially negative in the stroma (Figure 4H). Interestingly, bcl-2 (Figure 4I) and GATA-6 (Figure 4J) were positive with a stronger and more diffuse staining in the epithelium compared to the stroma. TLE-1 was diffusely positive in the epithelium and weak focal in the stroma (Figure 4K).

CDX-2, TTF-1, PAX-8, SALL4, ERG, WT-1, calretinin, SOX-10, S-100, CD10, b-catenin, NUT, AR, PR, chromogranin, PDX-1, POU2F3, MASH-1, GFAP, and NeuroD were all negative. P53 was wild-type. INI-1 and BRG-1 retained nuclear expression. Ki-67 showed an increased proliferation index (11.6%).

Targeted next-generation sequencing

Targeted next-generation sequencing (NGS) analysis of 161 genes was performed using the institutional Oncomine™ Comprehensive Assay v5.12 using an Ion Torrent™ NGS platform (ThermoFisher Scientific, Waltham, MA, US) according to the manufacturer’s instructions. A detailed list of genes tested is available online (https://mgp.upmc.com/Home/Print/Oncomine).

Oncomine testing was negative for DNA pathogenic variants, copy number alterations (CNAs), and gene fusions. The tumor was microsatellite-stable with a tumor mutational burden of <1 mutation/Mb. The tissue was subsequently sent for FoundationOne molecular testing, which did not detect any reportable genomic alterations.

RNA sequencing

RNA was isolated from the tumor sample according to established laboratory procedures. RNAseq libraries were prepared using Illumina RNA Prep with Enrichment (L) Tagmentation kit and sequenced on the NextSeq instrument. Data were analyzed using an in-house bioinformatics pipeline and three fusion detection software. Reads were aligned to the genome build hg19/GRCh37. Positive fusion calls were made by assessing unique in-frame sequencing reads spanning both fusion partners (chimeric reads) of known pathogenic fusions and fusions involving known cancer genes. The limit of detection for gene fusion was 10% of the input cells.

RNA sequencing identified an SS18::SSX4 fusion (5’ SS18 breakpoint corresponding to chr18:23612362 and 3’ SSX4 breakpoint corresponding to chrX:48248787).

Fluorescence in situ hybridization

Fluorescence in situ hybridization (FISH) for SS18(SYT) (Probe: LSI SS18(SYT) Dual-color Break-apart Probe 18q11.2; Abbott Molecular, Des Plaines, IL, US) was performed and targeted both the epithelial and stromal components, separately. The two probes are separated by approximately a 56 kb gap within the SS18(SYT) gene. SS18(SYT) FISH analysis was manually performed and quantitatively assessed by analysis of a minimum of 60 cells using the SS18(SYT) SpectrumOrange (telomeric, 5’ to SS18) and the SS18(SYT) SpectrumGreen (centromeric, 3’ to SS18) probes.

SS18(SYT) FISH studies were positive for translocation in both components (Figure 4D, inset, and Figure 4L, arrows). Interestingly, there was additional amplification of the 5’ signal (orange), more prominent in the epithelial component (Figure 4L, arrowheads), corresponding to the 5’ end of the SS18 region. FISH studies for EWSR-1 and FUS were also performed and were negative for rearrangements.

Overall, these findings were compatible with epithelial-predominant SS with neuroendocrine differentiation/transformation in the epithelial component. It was considered to have poorly differentiated features, and as per the Fédération Nationale des Centres de Lutte Contre le Cancer (FNCLCC), it best fit with grade 3.

Clinical follow-up

The patient completed radiation to the right inguinal region (35 Gray in five fractions), and the decision was to subsequently observe the patient. The patient developed right lower extremity lymphedema but showed no evidence of disease at five months postoperatively.

## Discussion

The monophasic spindle cell variant of SS is the most common SS subtype. On the other hand, epithelial-predominant SS is exceedingly rare. In our case, the solid areas comprised of sheets of ovoid cells with minimal cytoplasm, and “salt and pepper” nuclei morphologically overlap with small cell carcinoma or a poorly differentiated carcinoma with neuroendocrine differentiation. The stroma in our case was unusually predominantly less cellular and collagenous, in contrast to the more typical “blue” and cellular stroma found in biphasic SS, making the consideration of SS less obvious at first glance. The presence of a focal spindle cell component, albeit subtle, was a valuable diagnostic clue for considering SS in our differential diagnosis. In our case, although the background stroma was predominantly fibrotic, focal stromal areas harbored a mild increase in cellularity with a vague whirling arrangement, which prompted further investigation for SS. Such focal stromal changes would be particularly challenging to detect on FNA or biopsy samples given their limited sample nature and the possibility of sampling bias.

The neuroendocrine features morphologically seen in our case are further supported by positivity for neuroendocrine markers such as synaptophysin and INSM-1, making this an epithelial-predominant SS with a true neuroendocrine phenotype. The only other epithelial-predominant SS with “overwhelming” neuroendocrine differentiation present to date is the one reported by Chen et al. [4] in a 30-year-old male patient for whom a metastatic neuroendocrine carcinoma was similarly an initial consideration [4]. Unlike our case, the epithelial component showed papillary and cribriform architecture with mucin and was positive for synaptophysin, chromogranin, CD56, nestin, and Bcl-2. As their detection of SS18 rearrangement was based solely on SS18 break-apart FISH, data on the fusion partner were not available, and no amplification of the 5’ end of the SS18 gene was noted.

The other unusual morphologic finding in our case was the prominence of ossification. This feature is thought to impart a better prognosis [5-8] with a five-year survival of 82% [9], as compared to 25%-62% [7] for SS in general. Among the very few SS cases with extensive ossification and available molecular testing, including information on the SSX fusion partner subtype, only SSX1 [8,10] and/or SSX2 (one case with prominent needle-shaped mitochondrial calcifications seen ultrastructurally) [7] partners have been identified with no SSX4 fusion partner identified to date. Thus, the prominence of ossification in our case may have been compatible with the initial slow-growing nature of the tumor, while the epithelial neuroendocrine overgrowth signaled tumor progression, thus explaining the recent size increase.

In the current case, SS18::SSX4 fusion, the rarest fusion subtype [11-16], was identified and involved the “usual” site at codon 410 on SS18 and codon 111 on SSX4. SS18 gene, located on 18q11.2, encodes a subunit of the SWI/SNF (BAF) complex that is important for chromatin remodeling and gene expression [17]. Based on a systematic review/meta-analysis [18], specific partners have not been shown to affect prognosis in SS, with the caveat that SS18::SSX4 is regarded as a rare fusion and, thus, not very well-represented. Similarly, the morphologic spectrum of SS with SYT::SSX4 fusion is unclear; the few SS cases with SYT::SSX4 fusion described in the literature were of the monophasic spindle SS subtype [11,15].

Epithelial-predominant SS is a rare morphologic subtype that harbors prominent epithelial overgrowth, greatly overlapping with carcinoma. Given that <10% of SSs occur in patients > 60 years of age, the diagnosis of SS is often not considered in this age group [2], which partly accounts for the diagnostic challenges in our case as the tumor occurred in a 62-year-old female patient. Cases in older patients more often show poorly differentiated histology and develop at unusual locations when compared with younger age groups [2]. Positivity for CK7 and EMA, frequently encountered in SS [19-21], can lead to a misdiagnosis of carcinoma. In this case, given the positivity, albeit weak, for breast immunohistochemistry (IHC) markers such as GATA-3 and ER, a metastatic breast carcinoma was a consideration; however, TRPS-1 (a sensitive marker for breast cancer) was notably negative in the epithelial component, thus arguing against a metastatic breast carcinoma.

Aside from neuroendocrine tumors, a round blue cell tumor of the Ewing family may be a consideration, particularly given the unexpected NKX2.2 expression. NKX2.2 has high sensitivity and moderate specificity for Ewing sarcoma and is typically negative in SS, but it can be positive in a good proportion of neuroendocrine tumors, including small-cell carcinoma (80%) and well-differentiated neuroendocrine tumors (45%) [22]. The NKX2.2 positivity here is likely explained by the neuroendocrine differentiation.

Other diagnostic considerations would include biphasic sarcomas like glandular malignant peripheral nerve sheath tumor (MPNST), especially since TLE-1 can be expressed in MPNST, albeit in a weak fashion [23]. The matrix production, particularly its peripheral distribution, raised consideration for ossifying fibromyxoid tumors or even osteosarcoma, but these were excluded morphologically. Other less common considerations included gynecological (sex cord-stromal) tumors, mesothelioma (related to the canal of Nuck), and collision tumors. Aside from exclusionary morphologic and immunophenotypic features, the detection of SS18 rearrangement in both the epithelial and stromal components, by FISH, trumps all the aforementioned possibilities and confirms the diagnosis of SS.

We have demonstrated by FISH that SS18 is translocated in both the stromal and epithelial components, confirming the truly biphasic nature of this SS. More interestingly, the epithelial component showed extra signals corresponding to the amplification of the 5’ end of SS18. This amplification of the 5’ region of SS18 may in part explain the epithelial progression in this case. Of note, this amplification was only picked up by FISH as opposed to the other orthogonal methods we used. This can be explained by the fact that Oncomine has little coverage of 18q11, and our RNA sequencing molecular testing (RNAseq) is not designed to detect CNAs.

Per AACR GENIE, SS18 amplification is present in 0.16% of carcinoma cases, mostly adenocarcinomas [24]. SS18 amplification does not seem to be a well-elucidated phenomenon in SS. Multiple copies/signals of the SS18 gene by FISH have been reported in 15% of SS (15/101) cases in a study by Amary et al., with the intact nuclei of 14/15 of these cases showing more than 20% break-apart signals [12]. This is suggested to represent either polyploidy or multiple copies of variable length of chromosome 18 in the region of the SS18 gene. This finding has also been reported in another study by Panagopoulos et al., who found 18q to be the most commonly gained region (in five out of 19 primary SS with net gains and/or losses of chromosomal material) by karyotype [16]. Although no morphologic description was provided for these cases, it was noted that four out of five cases with a gain of 18q later gave rise to distant metastases [16]. The single case that harbored SS18::SSX4 fusion in their study did not, however, show a gain of 18q [16].

In our query of the neuroendocrine component [25], we found GATA-6 expression, which may be related to the 5' SS18 amplification. Human GATA-6 maps to 18q11.1-q11.2, which is in direct juxtaposition to SS18 and may be co-amplified in this case. It is notable that the GATA-6 expression is more prominent in the epithelial component, which overlaps with the 5' amplification localization. The role of GATA-6 in SS would need to be further evaluated, as it may have a role in mesoderm-derived sites and their related tumors [26,27]. GATA-6 (GATA-binding protein 6) has been previously demonstrated to be a marker of endocrine pancreatic development [28]. A study by Deng et al. [29] showed that GATA6 promotes epithelial-mesenchymal transition (EMT) through the MUC1/β-catenin pathway in cholangiocarcinoma. GATA6 also acts as an oncoprotein in gastric cancer, colorectal cancer, breast cancer, and cutaneous T-cell lymphoma whereby it promotes tumor progression [29].

Bcl-2 positivity, as assessed by IHC, is an almost general constitutive alteration of SS, although it is typically largely restricted to the spindle cell component, in contrast to the preferential expression in the epithelial component in our case [15,30]. BCL-2 (located on 18q21.33) is also in close proximity to SS18. While commonly overexpressed in SS, bcl-2 upregulation seems independent of amplification (or rearrangement) at the genomic level [21]. Further molecular studies are needed to elucidate the different implications and/or interactions of SYT::SSX1/2/4 and the role of BCL-2 in SS tumor onset and growth [15].

## Conclusions

We herein describe a unique epithelial-predominant SS case presenting in an uncommon age group, showing neuroendocrine transformation and extensive ossification and harboring SS18::SSX4 fusion, with SSX4 being a rare fusion partner. This is not only the second epithelial-predominant SS case with neuroendocrine differentiation to be reported, but one that gives probable insight into the pathogenesis of SS, particularly monophasic epithelial SS. Additionally, the presence of 5’ region amplification of SS18 is a novel finding that may be related to the neuroendocrine differentiation/transformation pathway of this rare tumor. Overall, epithelial-predominant SS with unusual neuroendocrine differentiation is a rare phenomenon and a potential diagnostic pitfall. It is important to recognize this uncommon SS variant and consider it in the differential diagnosis of metastatic carcinomas. Careful morphologic examination and thorough immunohistochemical and molecular ancillary testing are important diagnostic tools in reaching the right diagnosis and in ruling out other problematic mimickers.
